# Application of neutrophil to lymphocyte ratio in ankylosing spondylitis: Based on bibliometric and visualization analysis

**DOI:** 10.1097/MD.0000000000038364

**Published:** 2024-05-31

**Authors:** Cong Chengzhi, Liu Jian, Hu Yuedi, Li Yang, Chen Yiming, Huang Dan

**Affiliations:** a The First Affiliated Hospital of Anhui University of Chinese Medicine, Hefei, China; b Anhui University of Chinese Medicine, First Clinical Medical College, Hefei, China.

**Keywords:** ankylosing spondylitis, bibliometrics, neutrophil to lymphocyte ratio, visualization

## Abstract

Ankylosing spondylitis (AS) as a autoimmune disease involves inflammatory responses in the development of the disease, often causing changes in the neutrophil to lymphocyte ratio (NLR). In the past few decades, research on the relationship between NLR and AS has generally shown an upward trend. This study adopts the bibliometrics method to analyze the development trend, frontier, and hotspots of global research in this field in the past 2 decades. By searching for publications in the SCI-Expanded edition of the Web of Science Core Collection, the information of literature published between 2000 and 2023 is recorded. Based on the VOSviewer, CiteSpace and Excel, bibliometric analysis, and visualization analysis are conducted on the overall distribution of annual output, leading countries, active institutions, journals, authors, co-cited references, and keywords. Through retrieving and screening, a total of 1654 papers are obtained for analysis. In the past 2 decades, the number of publications related to this field has shown an increasing trend. The United States has the highest Hirsch index (H-index) and publication volume. The most productive institution is Harvard University, while the H-index of the University of Milan in Italy is far ahead. Frontiers in Immunology is the institution with the highest output. The H-index of the Annals of the Rheumatic holds the top position. This study has uncovered the main emphasis on NLR in AS research and has provided clarification regarding the value of NLR as a biomarker for immune inflammatory response in the diagnosis and prognosis of AS.

## 1. Introduction

Ankylosing spondylitis (AS), a type of spinal arthropathy, is in fact an autoimmune diseases, mainly affecting the spinal joints, sacroiliac joints, and adjacent soft tissues such as tendons and ligaments.^[1]^ The progression of the disease can lead to fibrosis and calcification, leading to loss of spinal flexibility and mobility with the potential for progressive spinal stiffness and inflammatory back pain, which can affect the gait ability of AS patients and cause inconvenience in movement.^[2]^ In addition, AS patients may also experience postural changes such as cervical flexion and decreased lumbar lordosis, as well as calcification of ligaments, intervertebral discs, and bone processes, and the incidence of cervical fractures in AS patients is higher than that in non-AS patients.^[3,4]^ It is reported that the most common emotional temperament of AS patients is anxiety and depression, which can increase disease activity and reduce the patient’s quality of life.^[5]^

Neutrophil to lymphocyte ratio (NLR) is a novel inflammatory marker that has been used in recent years for the diagnosis, prognosis, and severity assessment of various diseases.^[6–8]^ Neutrophils, as crucial immune cells in the human body, serve as essential mediators of inflammation, while lymphocytes play a key role in immune function within the immune system.^[9,10]^ Widely utilized in medical science, NLR is considered an evaluation index for systemic inflammatory response and has valuable implications in the diagnosis and prognosis evaluation of rheumatic diseases. Several retrospective clinical studies have demonstrated that blood NLR is a simple and readily accessible inflammatory marker, independent of other factors, and can predict the presence of rheumatic diseases^.[11–14]^ Notably, a comparison of NLR values between AS patients and healthy controls showed a statistically significant mean difference of 0.38 (95%CI: 0.24–0.52, *P* < .0001), thereby suggesting that higher NLR values correlate with underlying immunoinflammation in AS.^[15]^

Bibliometrics is an interdisciplinary field that combines mathematics, statistics, and philology to establish a comprehensive quantitative knowledge system. Utilizing information visualization technology, bibliometrics aims to visually uncover the research status, key areas of interest, and future trends in a particular field.^[16–21]^ However, no bibliometric analysis has been conducted on the AS-NLR research area to date. To address this knowledge gap, the primary objective of this study is to extensively investigate the current research status, prominent areas of interest, and emerging trends in this field. Additionally, this study aims to provide robust scientific data to support the validity of utilizing NLR as a biomarker for AS.

## 2. Materials and methods

### 2.1. Information sources and retrieval methods

The Web of Science Core Collection (WoSCC) database, renowned for its authoritative, up-to-date, and comprehensive data, has emerged as a vital resource for researchers worldwide seeking access to the latest scientific research findings and a deeper understanding of research developments.^[22]^ Notably, the Science Citation Index Expanded within WoSCC encompasses esteemed journals across various disciplines, including the natural sciences and biomedicine. Therefore, we have included this database as the primary source of data for our study.

**Figure 1.**
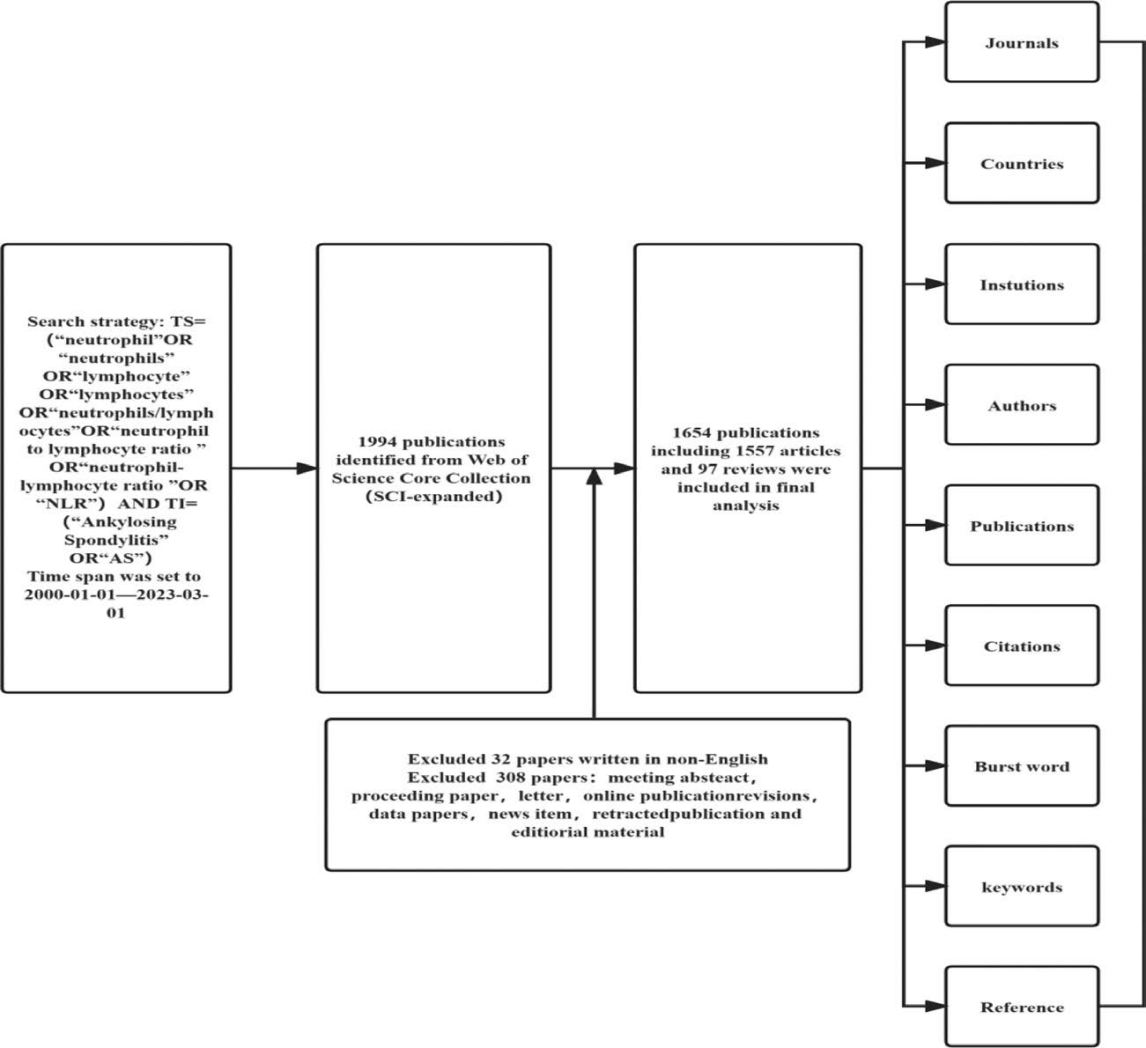
Flow chart.

Considering the rapid update of the database, the literature retrieval was conducted within 1 day to avoid deviation. The retrieval strategy is as follows: TS = (“neutrophil” OR “neutrophils” OR “lymphocyte” OR “lymphocytes” OR “neutrophils/lymphocytes” OR “neutrophil-lymphocyte ratio” OR “neutrophil to lymphocyte ratio” OR “NLR”) AND TI = (“ankylosing spondylitis” OR “AS”), from January 1, 2000 to March 1, 2023, language limited to English. A total of 1994 papers were retrieved, 2 experienced doctors imported the literature information (including titles, abstracts, and keywords) into Excel for meticulous data cleaning. Any discrepancies were resolved through discussion, resulting in the exclusion of 340 invalid literature entries. Ultimately, we obtained a final dataset comprising 1654 valid publications, consisting of 1557 research articles and 97 reviews. Following a second review conducted using Excel, we confirmed that there was no content unrelated to the topic present. The data sets included in the analysis were recorded and analyzed, taking into account various information such as author, institution, country, journal, number of citations, year of publication, Hirsch index (H-index), keywords, and references. The specific retrieval process is shown in Figure 1.

### 2.2. Synonym replacement

To mitigate potential bias caused by variations in country names, journals, authors, and keywords, we have taken measures to replace certain synonyms. Prior to data analysis, we, for instance, reclassified publications from Taiwan as belonging to China, and publications from England, Northern Ireland, and Scotland as part of the United Kingdom.

### 2.3. Bibliometrics analysis

Based on VOSviewer (Nees Jan van Eck and Ludo Waltman, Leiden University, Leiden), CiteSpace (Chaomei Chen, Drexel University, Philadelphia, PA) and Microsoft Excel 2019 the obtained data were incorporated into analysis. VOSviewer (version 1.6.19) can be used to generate various relationship spectrum based on bibliometrics for constructing and visualizing bibliometric network diagrams, such as the cooperation diagram of “nation,” “institution,” and “journal,” as well as the co-citation diagram of “reference,” thereby quickly sorting out relevant literature in this field.^[23]^ CiteSpace (version 6.2) is a software that can identify relevant trajectories of hot topics in a field, which can be used to focus on analyzing the most core knowledge field, such as the “burst keyword” diagram in this article that predicts future trends in the research field based on the intensity and duration of the burst.^[24]^ Excel effortlessly manages a substantial volume of data, encompassing tasks like data entry, screening, verification, and more. In this paper, the bibliographic information from the analyzed articles is seamlessly imported into Excel for meticulous review, further enhancing the data’s scientific credibility.

The important indicators used in this article included the total publication volume (TP), total number of citations of total publications (TC), average citation (AC), and H-index. These indicators can be used to quantify the output capacity and influence of a country or institution. The H-index has gained widespread use in evaluating researchers, as it accounts for both the quantity and quality of their academic output. It offers a more accurate reflection of the academic influence of individuals, institutions, or even countries.^[25,26]^ Furthermore, the application of Excel provides predictive capabilities in determining the annual count of published documents through the implementation of a polynomial model, enabling a more detailed analysis of its fluctuations. Specifically, the variable f(x) represents the number of studies per year, while the variable × denotes the corresponding year of publication.

### 2.4. Ethics

This article does not contain any studies with human participants or animals performed by any of the authors. The manuscript submitted does not contain information about medical device(s)/drugs. No funds were received in support of this work.

## 3. Results

### 3.1. Global publication output results and trends

**Figure 2.**
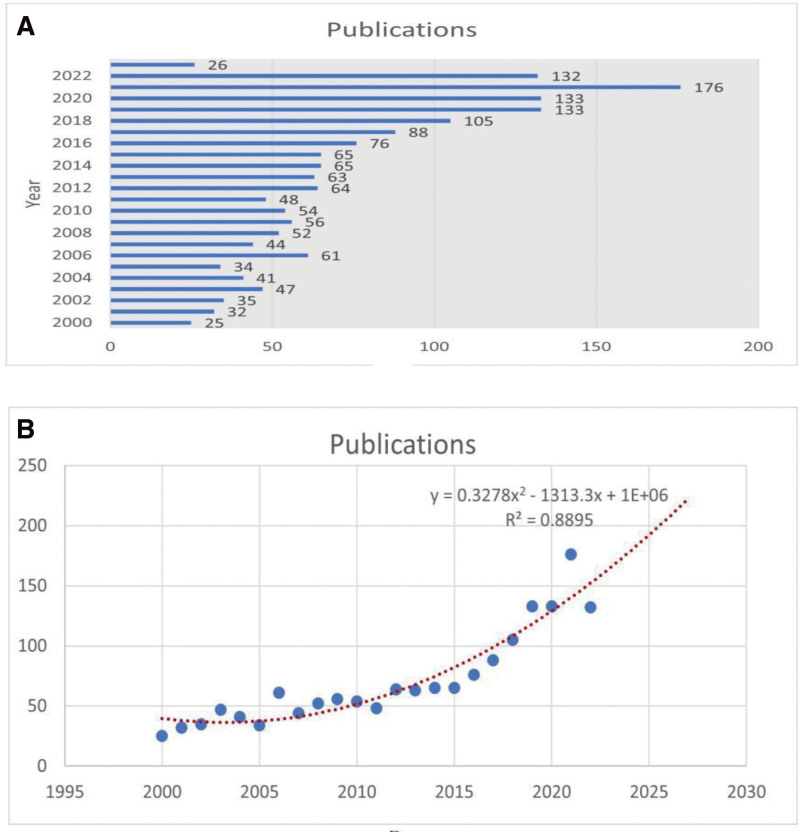
(A) Number of annual publications. (B) Curve fitting of the annual overall growth trend of publications. *Note*: The 1654 papers used in this study were by 6314 authors from 1968 institutions in 86 countries, published in 721 journals and contained 14,406 citations from 9469 journals.

A total of 1654 papers meet the inclusion criteria for the study, including 1557 research articles and 97 reviews. The annual circulation of publications relevant to the AS-NLR field is shown in Figure 2A. Since 2000, the overall annual publication volume in this field has remained stable at around 60 to 90 papers. After 2018, the publication volume has grown rapidly and reached a peak of 176 in 2021. It can be seen from the figure that although there are slight fluctuations in certain years, the overall annual publication volume in this field shows a steady upward trend. Figure 2B depicts a polynomial fitting curve of the annual overall growth trend of publications. According to the time curve, it is estimated that the global cumulative number of publications in this field will continue to rise in the next decade, with a correlation coefficient of R^2^ = 0.8895. These data indicate that the research on AS-NLR has always received widespread attention from scholars and will become a hot topic in the future, with rapid development.

### 3.2. Research status

#### 3.2.1. Country analysis

**Figure 3.**
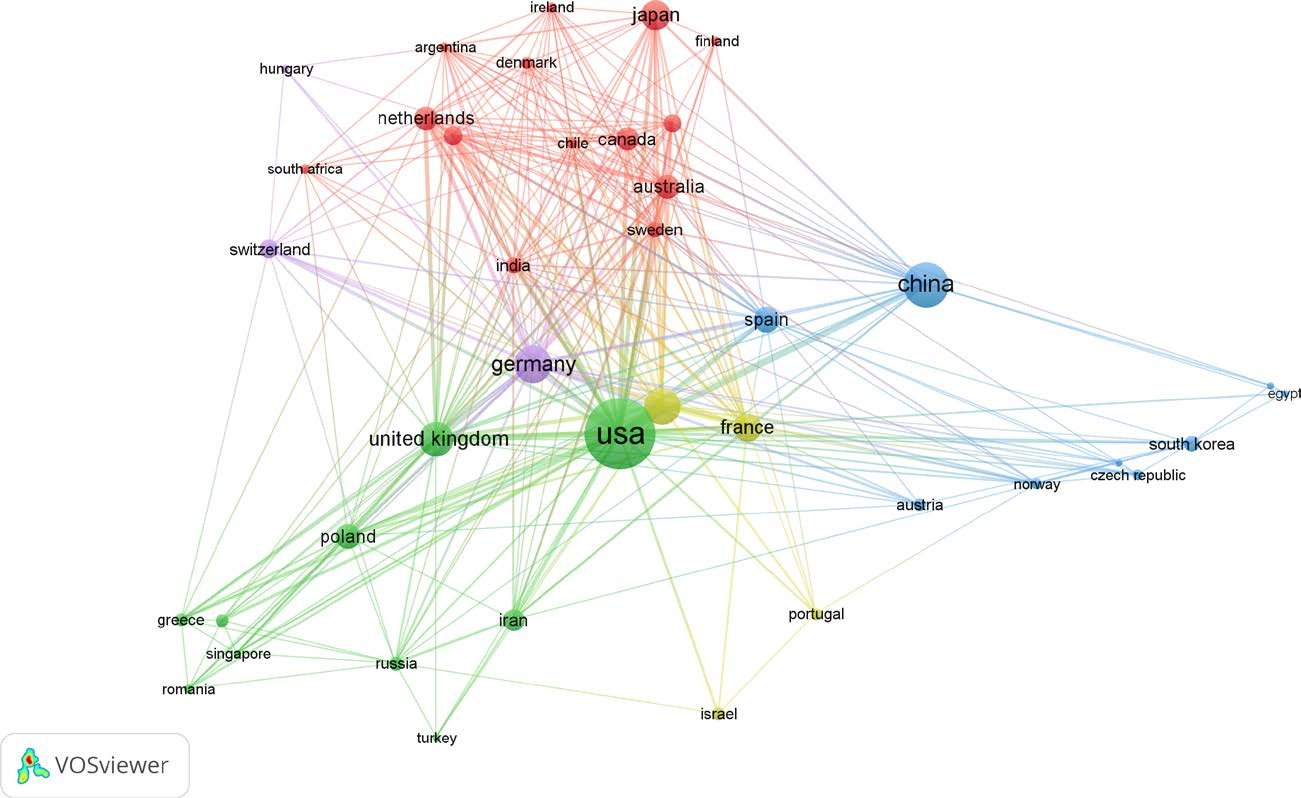
Cooperation networks between countries with a publication volume of ≥5.

By analyzing 86 countries and regions, The USA has the highest publication volume (TP), ranking first with 521 papers and accounting for 31.50% of the total, followed by China (198, 11.97%), Germany (142, 8.57%), Italy (139, 8.39%), England (100, 6.04%), Japan (92,5.55%), France (78, 4.71%), and Spain (68, 4.10%). Through visualization analysis of the evaluation indicators, the results show that the USA has the highest TC value (41,087), AC value (76.94), and H-index (95), and has a close cooperation relationship with other high-yield countries such as Germany, Italy, and England in this field. It is indicated that the USA attaches great importance to research in this field and possesses a certain degree of authority(Figure 3 and Table 1).

**Table 1:** Top 10 countries in global publication rankings.

Rank	Country	TP	% (n = 1654)	TC	AC	H-index
1	USA	521	31.50	41,087	78.86	95
2	China	198	11.97	3659	18.48	28
3	Germany	142	8.59	11,385	80.18	76
4	Italy	139	8.40	9677	69.62	61
5	England	100	6.05	6153	61.53	70
6	Japan	92	5.56	3175	34.51	26
7	France	78	4.72	3695	47.37	42
8	Spain	68	4.11	2811	41.34	19
9	Poland	66	3.99	2375	35.98	24
10	Netherlands	58	3.51	4276	73.72	39

AC = average citation, AC = TC/TP, TC = total number of citations of total publications, TP = total publication volume.

#### 3.2.2. Institution analysis

**Figure 4.**
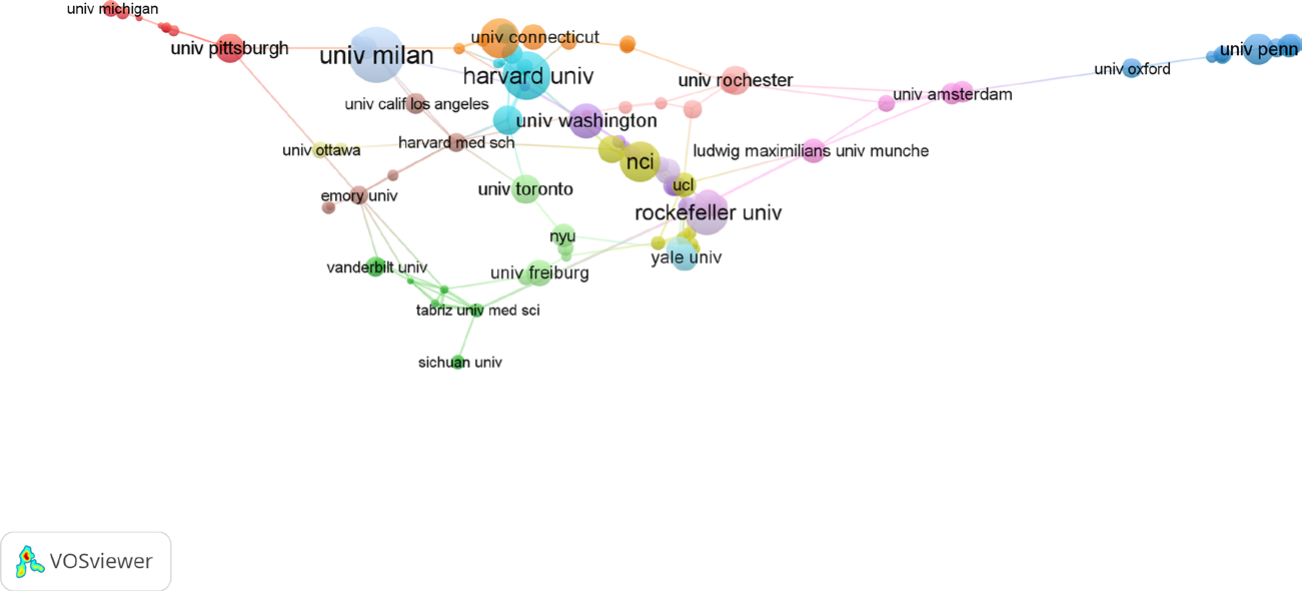
The cooperation network between institutions with a publication volume of ≥5 (the line between the 2 points in the figure indicates that the 2 institutions have established a cooperative relationship).

Institution analysis can quickly understand representative authoritative organizations in the relevant field. Table 2 lists the top 10 institutions with the highest publication volume. Harvard University has the highest TP and the highest productivity in this field, while Milan University occupies the leading position in AC and H-index. Among the top 10 institutions, the proportion of institutions from the United States is the highest, there is also relatively close cooperation between the country’s internal institutions. Figure 4 shows the cooperation network between institutions with the number of publications ≥ 5.

**Table 2:** The top 10 institutions with the number of publications.

Rank	Affiliations	Country	TP	TC	AC	H-index
1	Harvard University	USA	36	3177	88.25	32
2	University of Milan	Italy	16	3588	224.25	36
3	University of Toronto	Canada	14	983	70.21	28
4	University of Washington	USA	14	1446	103.29	22
5	Karolinska Institute	Sweden	13	881	67.77	29
6	French Institute of Medical Sciences	France	13	799	61.46	32
7	University College London	England	13	714	145.39	25
8	Tehran University	Germany	13	321	92.67	26
9	University of Pittsburgh	USA	12	1040	86.67	22
10	Tabriz Medical University	Iran	11	230	20.91	16

AC = average citation, AC = TC/TP, TC = total number of citations of total publications, TP = total publication volume.

#### 3.2.3. Journal analysis

Table 3 lists the publishing countries, TP, TC, H-index, and IF of the top 10 most productive journals. Frontiers in Immunology publishes the most papers in this field (87 papers, 5.26%). Frontiers in Immunology is an official journal of the International Union of Immunology (IUIS), focusing on clinical immunology and basic medicine. The Annals of Rheumatology achieves the highest H-index in this field, which serves as the top publication in the field of rheumatology and covers various aspects of rheumatology, with high quality control and influence in publication.

**Table 3:** Top 10 most influential journals.

Rank	Source	Country	TP	TC	AC	H-index	JCR	IF (2022)
1	Frontiers in Immunology	UK	87	2649	30.45	27	Q1	7.3
2	International Journal of Molecular	Swiz	51	1120	54.03	19	Q1	5.6
3	Journal of Leukocyte Biology	USA	28	2484	88.71	22	Q2	5.5
4	Annual Review of Immunology	USA	26	3543	136.27	32	Q1	29.7
5	Cancers	Swiz	19	309	16.26	20	Q2	5.2
6	Frontiers in Oncology	Italy	13	223	17.15	18	Q2	4.7
7	Annals of Rheumatism	UK	12	378	31.50	34	Q1	27.4
8	Rheumatology International	Germany	12	334	27.83	17	Q2	4.0
9	Clinical Rheumatology	UK	10	299	29.90	14	Q3	3.4
10	Journal of Immunology	USA	9	212	23.56	27	Q2	4.4

AC = average citation, AC = TC/TP, JCR = Journal Citation Reports Category, TC = total number of citations of total publications, TP = total publication volume.

#### 3.2.4. Author analysis

Table 4 shows the top 5 most productive authors. In terms of publication frequency, Jenne dieter and Korkmaz brice from the Technical University Munich are authors with the highest publication frequency (6 papers), followed by Mortrara lorenzo (5 papers) and Van Egmond marjolein (5 papers). In addition, Jenne dieter, and Korkmazbrice are also the authors with the highest TC, AC, and h-index. Based on a comprehensive literature review, the findings indicate a strong collaborative relationship between the 2 authors. Their primary research interests revolve around molecular mechanisms, clinical diagnosis, and various other aspects.

**Table 4:** Top 5 most productive authors.

Rank	Author	Country	Affiliations	TP	TC	AC	H-index
1	Jenne, Dieter	Germany	Technical University Munich	6	626	104.33	22
2	Korkmaz, Brice	Germany	Technical University Munich	6	626	104.33	22
3	Van Egmond, Marjolein	Netherlands	University of Amsterdam	5	196	39.2	9
4	Mortara, Lorenzo	Italy	University of Milan	5	166	33.2	18
5	Bruno, Antonino	Italy	University of Milan	4	165	41.25	20

AC = average citation, TC = total number of citations of total publications, TP = total publication volume.

In terms of their respective countries, 2 of the top 5 authors are from German institutions and 2 are from Italian institutions, indicating that Germany and Italy have more outstanding research scholars in this field.

### 3.3. Bibliographic coupling analysis

#### 3.3.1. Country/region

**Figure 5.**
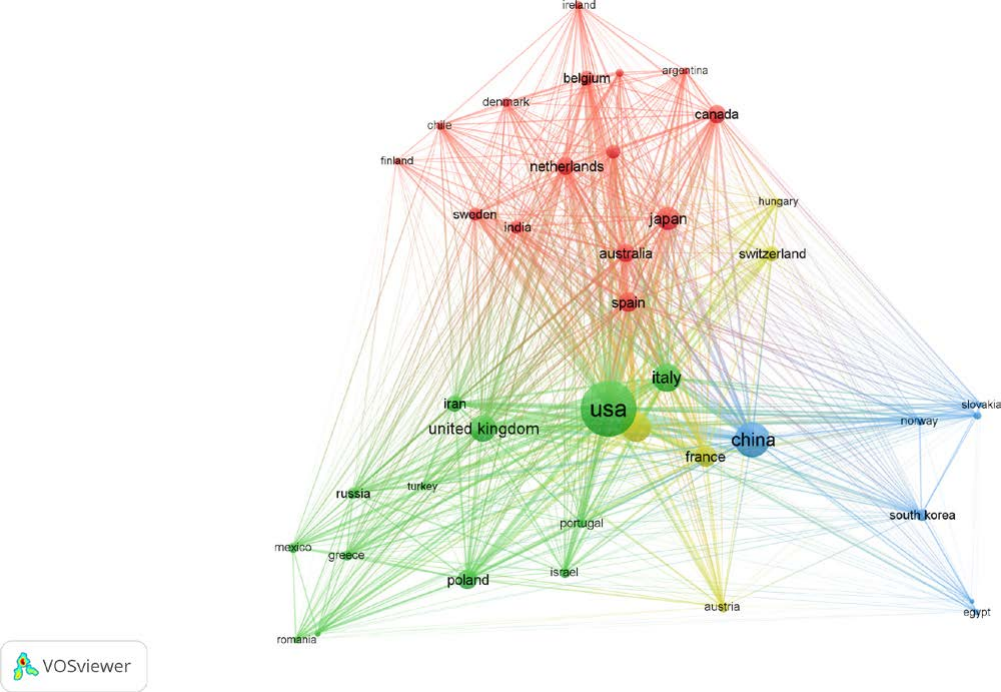
Bibliographic coupling analysis of global research on AS-NLR. Distribution diagram of 43 countries in terms of AS-NLR. The line connecting 2 points in the figure indicates that 2 countries have established a similar relationship. The thicker the line, the stronger the similarity between 2 countries.

Bibliographic coupling is a method that uses citation analysis to establish a comparison of similar relationships between documents, indicating that 2 publications share a common theme. Here we define that each selected country/institution/journal has at least 5 papers. The article data included in the analysis were imported into VOSviewer, resulting in a total of 43 countries cited in the articles, and the top 5 countries in the total link strength are the USA (72,151 times), Germany (11,385 times), Italy (9677 times), England (6153 times), and China (3659 times) (Fig. 5).

#### 3.3.2. Institution

**Figure 6.**
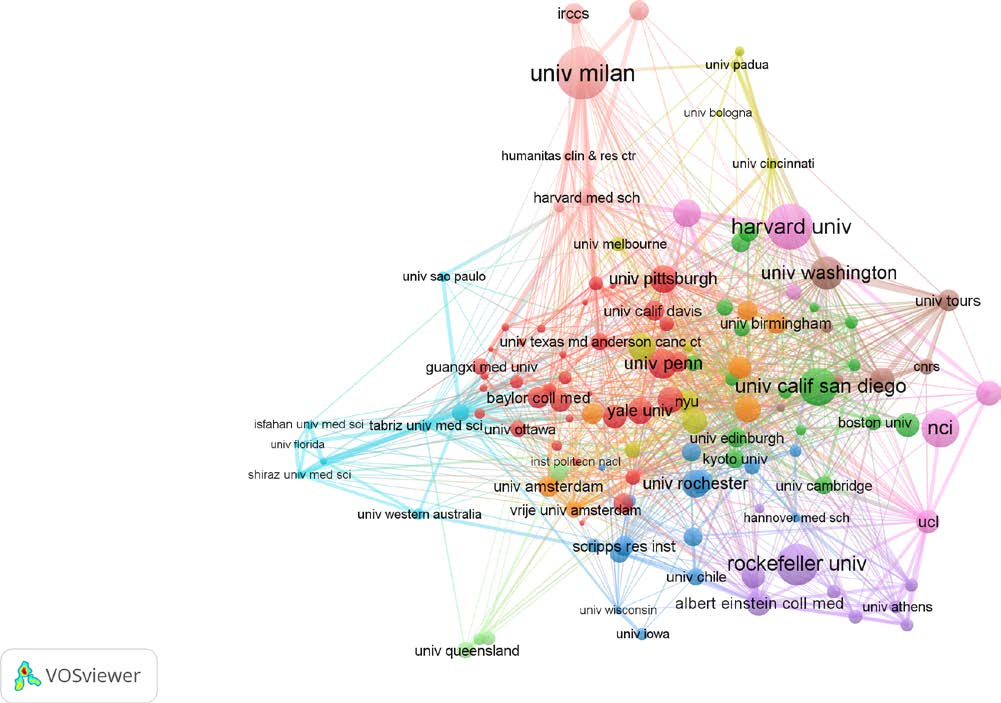
Mapping of 123 AS-NLR institutions. The line connecting 2 points in the figure indicates that 2 institutions have established a similar relationship. The thicker the line, the stronger the similarity between 2 institutions.

123 institutions were included in the analysis, the top 5 institutions in terms of total link strength are Harvard University (4733 times), University of Milan (3430 times), Ludwin University in Munich (2577 times), University of London (2546 times), and Karolinska College (2350 times) (Fig. 6).

#### 3.3.3. Journal

**Figure 7.**
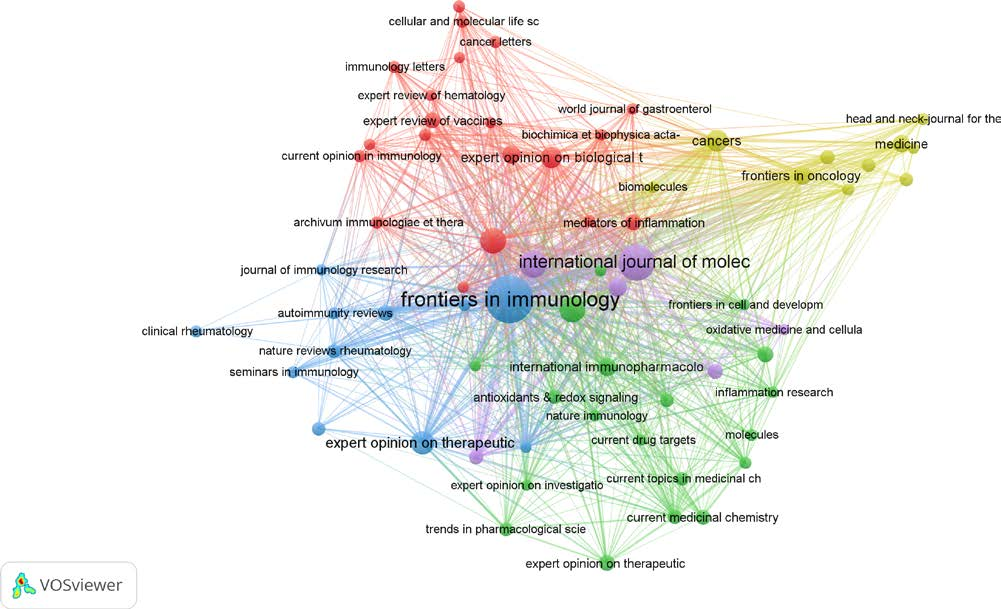
Mapping of 66 identified AS-NLR journals. The line connecting 2 points in the figure indicates that 2 journals have established a similar relationship. The thicker the line, the stronger the similarity between 2 journals.

Sixty-six journals were included in the analysis, the top 5 journals in total link strength are Frontiers in Immunology (4845 times), International Journal of Molecular (2623 times), Journal of Leukocyte Biology (1909 times), Annual Review of Immunology (1664 times), and Cancers (1199 times) (Fig. 7).

### 3.4. Analysis of co-cited references

**Figure 8.**
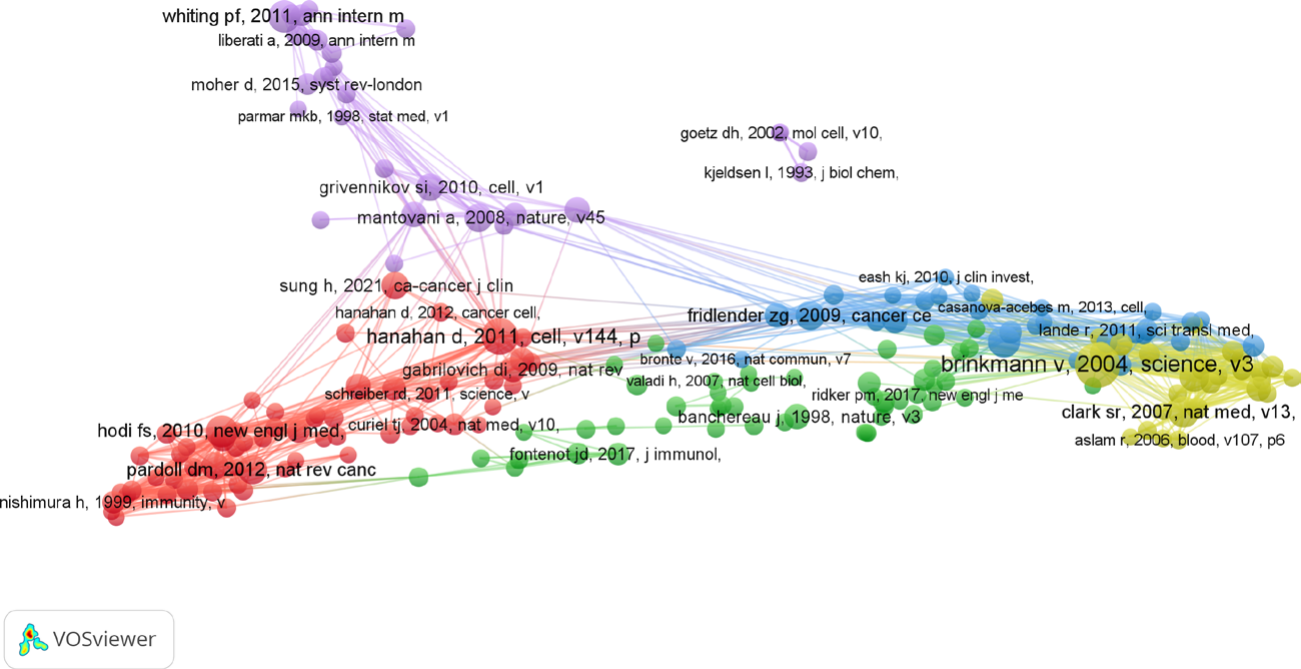
Mapping of 198 references with a minimum of 10 citations.

Co-cited references refer to 2 or more references being co-cited by one or more subsequent papers, which can quickly understand the research status in the field and the common concerns of similar literature. Given the large number of references in this study, 198 references (with a minimum of 10 citations) are selected to draw a co-cited analysis network. A line connecting 2 nodes indicates that they are referenced in the same publication, while a shorter line indicates a closer connection between the 2 publications. In addition, different color clusters represent different research topics (Fig. 8). The first cluster (red) aims to explore the mechanism between neutrophils and immune inflammation; the second cluster (green) focuses on exploring the immune mechanisms and pathogenic pathology of AS and interleukin (1L-17); the third cluster (blue) mainly focuses on the expression of cytokines related to AS and neutrophil lymphocytes; the fourth cluster (yellow) explores the mechanism between the formation of neutrophil extracellular traps (NETs) and AS; the fifth cluster (purple) is mainly related to the mechanism of T cell immune response. Based on clustering, it is found that most papers emphasize the immune mechanisms of AS and new therapeutic targets related to lymphocytes. Table 5 lists the top 10 references with the highest frequency of co-citations.

**Table 5:** Top 10 references with the highest number of co-citations.

Rank	Co-cited references	TC
1	Brinkmann, V, Reichard, U, Goosmann, C, et al. Neutrophil extracellular traps kill bacteria. Science. 2004;303(5663):1532–5.^[27]^	71
2	Hanahan, D, Weinberg, RA. Hallmarks of cancer: the next generation. CELL. 2011;144(5):646–74.	47
3	Fridlender, ZG, Sun, J, Kim, S, et al. Polarization of tumor-associated neutrophil phenotype by TGF-beta: “N1” versus “N2” TAN. Cancer Cell. 2009;16(3):183–94.	30
4	Fuchs, TA, Brill, A, Duerschmied, D, et al. Extracellular DNA traps promote thrombosis. P Natl Acad Sci USA. 2010;107(36):15880–5.^[28]^	30
5	Hodi, FS, O’Day, SJ, McDermott, DF, et al. Improved survival with ipilimumab in patients with metastatic melanoma. New Engl J Med. 2010;363(8):711–23.	35
6	Clark, SR, Ma, AC, Tavener, SA, et al. Platelet TLR4 activates neutrophil extracellular traps to ensnare bacteria in septic blood. Nat Med. 2007;13(4):463–9.	33
7	Topalian, SL, Hodi, FS, Brahmer, JR, et al. Safety, activity, and immune correlates of anti-PD-1 antibody in cancer. New Engl J Med. 2012;366(26):2443–54.	25
8	Kolaczkowska, E, Kubes, P. Neutrophil recruitment and function in health and inflammation. Nat Rev Immunol. 2013;13(3):159–75.^[29]^	35
9	Mantovani, A, Cassatella, MA, Costantini, C, et al Neutrophils in the activation and regulation of innate and adaptive immunity. Nat Rev Immunol. 2011;11(8):519–31.	22
10	Warnatsch, A, Ioannou, M, Wang, Q, et al Inflammation. Neutrophil extracellular traps license macrophages for cytokine production in atherosclerosis. Science. 2015;349(6245):316–20.	16

TC = total number of citations of total publications.

### 3.5. Analysis of highly cited references

Among the 1654 literature obtained, 198 reach the threshold of minimum citation frequency ≥ 10 (Fig. 8). Table 6 summarizes detailed information on the top 10 most frequently cited publications on AS-NLR, mainly published from 2004 to 2015. One of the most frequently referenced papers in the field is a study titled “Neutrophil extracellular traps kill bacteria,” which was published in the esteemed journal Science. This paper validated the role of NETs as a form of innate response in the treatment of immune inflammatory disease AS through cell and animal experiments, and confirmed the key contribution of NETs in the immune pathogenesis of AS.^[27]^ In addition, a review titled “Extracellular DNA traps promote thrombosis” by Fuchs Tobias A. in 2010 also explored the mechanism between NETs and thrombosis in immune inflammatory diseases, further demonstrating the close relationship between NETs and AS.^[28]^ It is worth mentioning that Kolaczkowska Elzbieta published a paper titled “Neutrophil recruitment and function in health and inflammation” in Nature Reviews Immunology and elucidated the mechanism between neutrophils and immune inflammatory diseases, confirming that neutrophils participate in inflammatory responses through T cell recruitment.^[29]^ This information suggests that NETs have garnered significant attention in the field of AS-NLR, highlighting their importance as a noteworthy biological phenomenon.

**Table 6:** Detailed information on the top 10 most frequently cited publications.

Rank	Title	First Author	Journal	JIF2022	JCR	Publication year	TC
1	Neutrophil extracellular traps kill bacteria	Brinkmann, V^[27]^	Science	56.9	Q1	2004	71
2	Hallmarks of cancer: the next generation s	Hanahan, D	Cell	64.5	Q1	2011	47
3	Polarization of Tumor-Associated Neutrophil Phenotype by TGF-beta: “N1” versus “N2” TAN	Fridlender, ZG	Cancer Cell	50.3	Q1	2004	30
4	Extracellular DNA traps promote thrombosis	Fuchs, TA^[28]^	Proceedings of The National Academy of Sciences of The United States of America	11.1	Q1	2010	30
5	Improved Survival with Ipilimumab in Patients with Metastatic Melanoma	Hodi, FS	New England Journal of Medicine	158.5	Q1	2010	35
6	Platelet TLR4 activates neutrophil extracellular traps to ensnare bacteria in septic blood	Clark, SR	Nature Medicine	82.9	Q1	2007	33
7	Safety, Activity, and Immune Correlates of Anti-PD-1 Antibody in Cancer	Topalian, SL	New England Journal of Medicine	158.5	Q1	2012	25
8	Neutrophil recruitment and function in health and inflammation	Kolaczkowska, E^[29]^	Nature Reviews Immunology	100.3	Q1	2013	35
9	Neutrophils in the activation and regulation of innate and adaptive immunity	Mantovani, A	Nature Reviews Immunology	100.3	Q1	2011	22
10	Neutrophil extracellular traps license macrophages for cytokine production in atherosclerosis	Warnatsch, A	Science	56.9	Q1	2015	16

TC = total number of citations of total publications, JCR = Journal Citation Reports Category.

### 3.6. Keyword co-occurrence, cluster analysis, and burst keyword analysis

**Figure 9.**
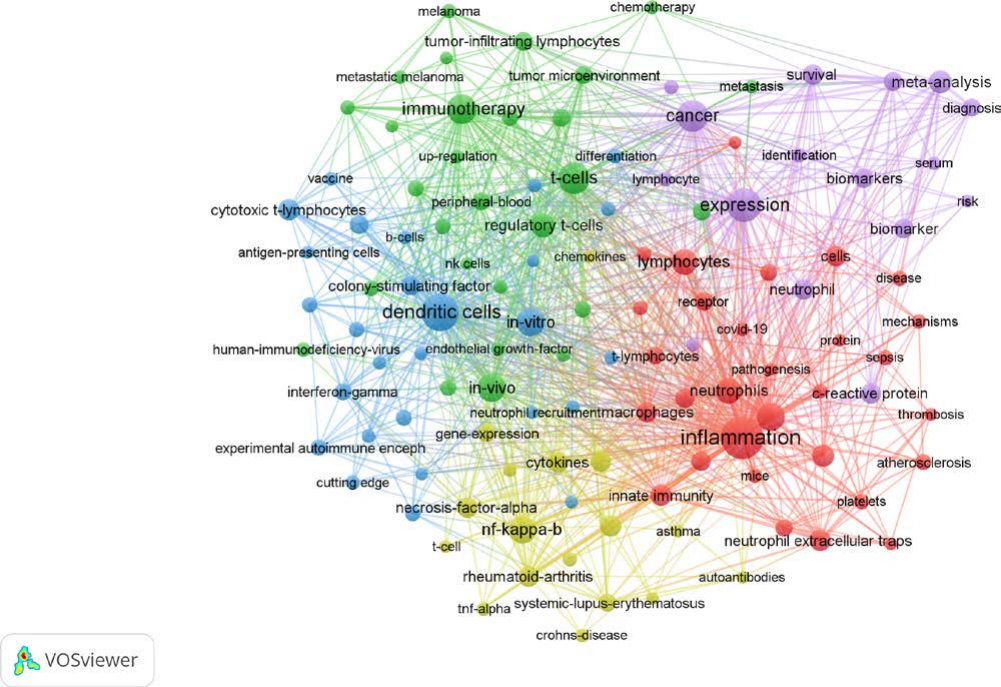
Co-occurrence clustering analysis of keywords in AS-NLR publications (co-occurrence frequency ≥ 20).

**Figure 10.**
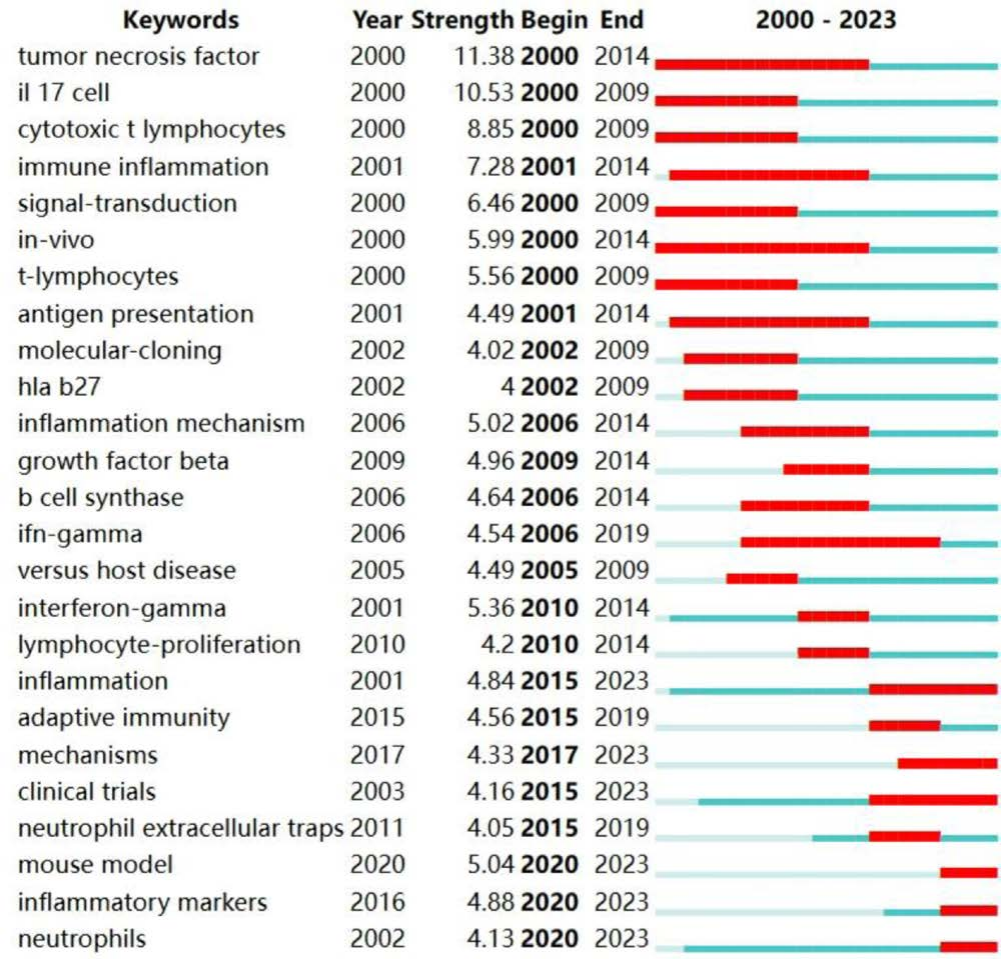
The top 25 keywords with strongest burst from 2000 to 2023. The year between “begin” and “end” represents the period when the keyword is more influential. The year in light green means that the keyword has not yet appeared; the year in dark green means that the keyword has less influence and the year in red means that the keyword has more influence.

In VOSviewer, “All keywords” is selected, and the keywords with the minimum co-occurrence of 20 are screened out. Finally, 118 keyword networks are obtained from 10,549 keywords (Fig. 9). In Figures 9, 118 keywords are divided into 5 different clusters. The red cluster mainly refers to the “pathogenesis,” with the main keywords being lymphocyte, inflammation, activation, etc. The main focus of the green cluster is “molecular research,” with the main keywords being t-cells, immunotherapy, CD8 (+) cells, etc. The blue cluster mainly highlights “clinical trials,” with the main keywords being in vivo, dentric cells, cyclic t-lymphocytes, etc. The yellow cluster focuses on “pathway factors,” with the main keywords being nf-kappa b, gene expression, rheumatoid arteritis, etc. The purple cluster primarily explores the “clinical mechanism,” with the main keywords being b lymphocyte, biomarker, protein, etc. Based on the analysis conducted using Citespace software, a keyword burst analysis was performed on 1654 collected literatures (Fig. 10). Among them, “tumor necrosis factor” and “in vivo” (2000–2014) signify the significant attention paid by scholars in this field to in vivo experiments and the expression of tumor necrosis factor over an extended period. The top 3 prominent factors include tumor necrosis factor (11.38), il17 cell (10.53), and cytotoxic t lymphocytes (8.85), highlighting the importance of cytokine studies in this field. In the last 3 years, the explosive keywords primarily revolved around “mouse model,” “inflammatory markers,” and “neutrophils,” suggesting that current research focus within this field centers on in vivo animal experiments aimed at validating inflammatory markers of AS and providing a scientific basis for its diagnosis and prognosis.

## 4. Discussion

This study represents the inaugural bibliometric analysis of AS-NLR research, utilizing the SCI-Expanded Science Citation Index (WoSCC) to survey a total of 1557 research articles and 97 reviews published from 2000 to 2023. The findings from the polynomial fitting curve analysis reveal a slight fluctuation in the publication count over the past 2 decades, with a consistent and upward trajectory overall. This observation signifies that scholars are increasingly taking interest in this field, indicating a promising future for research in AS-NLR.

Using VOSviewer, the selected dataset was imported into the software for comprehensive analysis, providing a visual representation of the research landscape in the field of AS-NLR across various dimensions such as countries, institutions, journals, and authors. The results indicate that the top 3 countries in terms of research contributions are the United States (521, 31.50%), China (198, 11.97%), and Germany (142, 8.57%). In the state-to-country cooperation network diagram, developed nations exhibit closer collaboration, reflecting earlier-established partnerships. It is noteworthy that the United States holds a prominent position of authority in this field. Regarding institutions, Harvard University emerges as the leading publisher of papers, while the University of Milan, despite publishing fewer papers than Harvard, boasts a superior AC value (224.25) and H-index (36), illustrating the high caliber and international recognition of the journals affiliated with the University of Milan. The analysis of journals reveals that Frontiers in Immunology is the most prolific in the AS-NLR field, while the Annual Review of Immunology achieves the highest AC value (136.27). The Annals of Rheumatism garners significant authority and influence in this domain, as evidenced by its highest H-index (34). These findings suggest that scholars in the AS-NLR field exhibit a strong inclination towards journals focused on rheumatic immunity and molecular mechanisms. In terms of individual scholars, German researchers Jenne, Dieter, and Korkmaz, Brice demonstrate close collaboration and wield considerable influence in the AS-NLR field. Their primary research interests lie in inflammatory disease cytokines and clinical diagnosis, establishing a foundation for investigating the potential molecular mechanisms of AS-NLR.

Subsequently, a bibliographic coupling analysis was conducted to elucidate the interconnectedness of publications within the field, focusing on country, institution, and journal relationships. The findings revealed that the United States exhibited the strongest linking strength, with Harvard University standing out as the most influential institution. Notably, Frontiers in immunology emerged as the journal with the highest degree of coupling intensity within this domain. These results further confirm the robustness of our previous findings.

Highly cited literature analysis facilitates a swift comprehension of the core research in this field. The most frequently cited works primarily address the role of NETs as an innate response in the treatment of immunoinflammatory disease, thereby corroborating the association between NETs and the aforementioned disease. Subsequently, based on keyword co-occurrence, cluster analysis, and keyword outbreak. Different colored clusters symbolize distinct research clusters. Over time, the study shifted from “clinical trial” and “molecular research” towards “clinical mechanism,” “cytokine,” and “pathogenesis.” Notably, the top 3 emerging keywords represent the most prominent cytokines in the field of AS-NLR research. Tumor necrosis factor (TNF), a small protein secreted by macrophages, acts as a bioactive anti-inflammatory factor among numerous cytokines, and plays a pivotal role in the systemic inflammatory response.^[30,31]^ Studies indicate that the expression levels of TNF-α can serve as a benchmark for evaluating the inflammatory response of AS.^[32]^ Interleukin-17 (IL-17) is a vital cytokine that regulates inflammation in immune responses, triggering various inflammatory cytokines such as TNF-α and IL-6. It exhibits a close connection with the onset and progression of rheumatic diseases.^[33–35]^ Cytotoxic T lymphocytes (CD8 + T cells) are a crucial component of the adaptive immune system, evolving from lymphocytes and becoming a prime target of the body’s immune defense through binding with effector target cells.^[36,37]^

NLR is a novel inflammatory indicator reflecting the dynamic balance between neutrophils and lymphocytes in peripheral blood. As a biomarker of immune inflammation diseases, it has garnered significant attention and has become a valuable and reliable method for monitoring systemic inflammatory diseases. Furthermore, it can serve as a marker for systemic rheumatism, as indicated by recent research.^[38–40]^ Meacan et al^[41]^ found that an increase in neutrophil count is generally associated with poor prognosis and increased mortality. A clinical meta-analysis study also showed that NLR is a reasonable measure for detecting systemic inflammation in AS patients and can indicate disease activity in AS patients.^[42]^

Our study, utilizing bibliometrics analysis, has identified the current frontiers and hotspots in AS-NLR research. These findings not only provide literature and data support for considering NLR as a biomarker of immune inflammation in AS but also shed light on the most prevailing topics immune inflammation and molecular mechanisms in this field. These insights lay a solid foundation for our future clinical research endeavors.

## Author contributions

**Data curation:** Cong Chengzhi.

**Formal analysis:** Cong Chengzhi.

**Investigation:** Liu Jian.

**Methodology:** Liu Jian.

**Supervision:** Hu Yuedi.

**Software:** Chen Yiming.

**Validation:** Li Yang.

**Visualization:** Huang Dan.

**Writing – review & editing:** Cong Chengzhi.
